# Accurate and Robust Floor Positioning in Complex Indoor Environments

**DOI:** 10.3390/s20092698

**Published:** 2020-05-09

**Authors:** Jingyu Huang, Haiyong Luo, Wenhua Shao, Fang Zhao, Shuo Yan

**Affiliations:** 1 School of Software Engineering, Beijing University of Posts and Telecommunications, Beijing 100876, China; jingyuhuang@bupt.edu.cn (J.H.); shaowenhua@ict.ac.cn (W.S.); zfsse@bupt.edu.cn (F.Z.); lightning@bupt.edu.cn (S.Y.); 2 Beijing Key Laboratory of Mobile Computing and Pervasive Device, Institute of Computing Technology Chinese Academy of Sciences, Beijing 200190, China

**Keywords:** floor positioning, Wi-Fi, barometric pressure, motion detection, HMM

## Abstract

With the widespread development of location-based services, the demand for accurate indoor positioning is getting more and more urgent. Floor positioning, as a prerequisite for indoor positioning in multi-story buildings, is particularly important. Though lots of work has been done on floor positioning, the existing studies on floor positioning in complex multi-story buildings with large hollow areas through multiple floors still cannot meet the application requirements because of low accuracy and robustness. To obtain accurate and robust floor estimation in complex multi-story buildings, we propose a novel floor positioning method, which combines the Wi-Fi based floor positioning (BWFP), the barometric pressure-based floor positioning (BPFP) with HMM and the XGBoost based user motion detection. Extensive experiments show that using our proposed method can achieve 99.2% accuracy, which outperforms other state-of-the-art floor estimation methods.

## 1. Introduction

Recently, more and more huge multi-story buildings have mushroomed, such as gymnasiums, super malls, convention centers, etc. To promote security supervision levels and facilitate the movement of people around complex indoor environments, accurate indoor positioning is getting more and more urgent. When an earthquake or fire occurs various complex indoor environmental factors, e.g., smoke and rubble, can increase rescue difficulties dramatically [1]. In this emergency case, indoor positioning can help rescuers plan a safe and reasonable route [2,3,4]. Nowadays, smartphones are not only communication tools but also measuring instruments embedded with various sensors [5], which can be used as a reasonable positioning terminal. Though there are many studies on indoor positioning, as an important prerequisite for indoor positioning in multi-story buildings, accurate and robust floor positioning remains a big challenge, especially in the multi-story buildings with large complex hollow areas through multiple floors. In practical applications, floor positioning is useful and plays an important role in indoor localization, which not only can reduce the location search space, but also can improve the positioning accuracy.

Current floor positioning methods are mainly classified into Wi-Fi-based methods, pressure-based methods and hybrid methods. Wi-Fi-based methods stem from Wi-Fi signal propagation characteristics. Wi-Fi signals decay rapidly when passing through concrete floor slabs from other floors. On the contrary, the Wi-Fi signals attenuate gradually when propagating without going through concrete walls on the same floor [6]. Such a propagation attenuation pattern leads to different sets of APs received on different floors and distinct Received Signal Strength Indicators (RSSIs) for the same AP, which makes it possible to identify floors according to the Wi-Fi fingerprints measured by smartphones.

However, smartphones situated in adjacent floors are prone to measure similar Wi-Fi fingerprints in the multi-story buildings which contain large hollow areas. Without the large attenuation generated by concrete floor slabs, Wi-Fi based methods cannot obtain accurate floor estimations in the hollow areas. Inspired by the fundamental notion that the barometric pressure is inversely proportional to altitude [7], as an alternative method, some studies have introduced the barometers widely embedded in modern smartphones (such as the Xiaomi 3/4/5, Samsung S4/NOTE2/3/4, Huawei Mate 7/8/9, Apple iPhone 6, etc.) to identify floors. However, due to the fact that the barometric pressure is easily affected by weather conditions and ambient air flows, barometric pressure measurements [8] cannot be used to identify floor levels directly without calibration with a standard barometer deployed in the target building, which has poor scalability in practice.

More recently, many studies have attempted to solve the floor positioning problem in complex multi-story buildings by combining Wi-Fi based methods and pressure-based methods [9,10,11]. The key to the hybrid methods is how and when to fuse the observations from different sensors. We propose a succinct and accurate fusion method by introducing a confidence parameter, which can take advantage of the complementary nature of Wi-Fi fingerprints and barometer measurements. For convenience of description, we use BWFP to represent our proposed Wi-Fi based floor positioning and BPFP to represent pressure-based floor positioning. Taking advantage of the natural complementarity of IMU/Wi-Fi/barometer while floor positioning, we propose a multimodal fusion solution for floor positioning to deal with the problem of low accuracy and poor robustness. In a nutshell, we utilize “reference pressure” and confidence as a bridge to integrate Wi-Fi based methods and pressure-based methods. We introduce an XGBoost-based floor switching behavior detection method to improve floor positioning and provide continuous vertical coordinates when floor switching behavior is being detected. 

The major contributions of the paper may be summarized as follows:

1. We propose a BWFP augmentation method with BPFP using HMM. In areas where Wi-Fi signals are highly distinguishable, BWFP is applied and the high-confidence floor estimation result is used to provide a reference value for a barometer. In the hollow areas with low discrimination for Wi-Fi signal, BPFP is applied by the mapping between the barometric pressure and altitude. HMM is utilized to correct the occasional floor positioning errors caused by the BWFP or the BPFP method.
2. We propose a motion detection method to identify user’s floor switching behavior based on accelerometer and gyroscope readings. Once the floor switching motion is detected, the vertical coordinates are estimated. Under this floor switching periods, location-based services can still be provided in the intermediate areas between adjacent floors.
3. We model the floor positioning into a supervised multi-classification problem and use the XGBoost [12] to make advantage of the received signal strengths for accurate floor positioning. Through combining multiple tree models, the XGBoost-based floor positioning method (i.e., BWFP) can obtain high-confidence floor positioning result in the closed areas. To speed up training of XGBoost, we introduce exponential preprocess for all Wi-Fi fingerprints.
4. We evaluate our proposed algorithm in several different scenarios and the experimental results demonstrate that our proposed algorithm outperforms the Wi-Fi-only-based method, the pressure-only-based method and the comparative hybrid method with 99.2% average accuracy and better robustness. Furthermore, the probability of floor switching detection delay within 2 s exceeds 90%, which is a vital metric for the floor positioning method.

The rest of this paper is organized as follows: In Section 2, related works are introduced. The materials and methods are described in Section 3. The experimental results are shown in Section 4. Finally, the discussion and conclusions of this paper are presented in Section 5.

## 2. Related Works

Many studies on floor positioning has been conducted in the past decades, which can be broadly classified into Wi-Fi-based methods, barometric pressure-based methods and hybrid methods.

### 2.1. Wi-Fi-Based Methods

Wi-Fi based methods, which integrate collected Wi-Fi fingerprints for floor positioning, are the most popular methods. Alsehly et al. [13] designed a group variance algorithm. It groups the variances of RSSI values in each floor. During the prediction phase, it compares the variance of newly-collected Wi-Fi signals with each group to find the best matching floor level. Deng et al. [14] attempted to use a k-means clustering algorithm for floor number identification. Campos et al. [15] employ the majority voting committees of back-propagation artificial neural networks (ANNs) to identify floors. It first clusters the collected RSS levels by unsupervised learning, and subsequently utilizes ANNs to identify the cluster of the newly-collected RSS values. FloorLoc-SL [16] introduced a self-learning scheme to create fingerprints for each floor. It proposes the use of an activity classification module and a floor counting module to detect the traces of users. When the received characteristic Wi-Fi signal cannot be found in the fingerprint database, the current floor will be given by the floor counting module and the new characteristic Wi-Fi signal will be added into the fingerprint database with the floor estimation. Sun et al. [17] propose a multi-floor localization framework and apply Fisher’s linear discriminant method to maximize the margin discrepancy between neighboring floors in fingerprint space. Stair walking and elevator taking event recognition trigger the floor estimation. F-Loc [11] collects and clusters the Wi-Fi fingerprints near the entrances of elevators and identifies the floor by detecting the taking elevator action using accelerometer readings. Li et al. [18] proposed a wireless received signal strength-profile-based floor-detection approach to enhance RSS-based floor detection performance. By calculating the average dilution of precision of successive Wi-Fi signals from different APs on each floor when users walk, the floor with the smallest positional DOP value is regarded as the floor estimation. Kim et al. [19] extend the single-input and multi-output deep neural network to enable hybrid building/floor classification and location coordinate regression with Wi-Fi fingerprints. This method exploits the hierarchical and different nature of the estimation of building/floor and floor-level location coordinates. Though many works have been conducted on the floor positioning with Wi-Fi signals, the existing Wi-Fi-based floor positioning methods still cannot accurately discriminate adjacent floors in the multi-story buildings which contain complex and large hollow areas.

### 2.2. Barometric Pressure-Based Methods

Without the large attenuation generated by concrete floor slabs in the larger hollow areas, smartphones situated in adjacent floors are prone to measure similar Wi-Fi fingerprints in multi-story buildings, which leads to wrong floor estimations by Wi-Fi-based methods. Some studies have proposed barometric altimetry for floor determination. However, barometric pressure is easily affected by weather conditions and ambient air flows. Muralidharan et al. [20] observed that although the absolute barometric pressure on each floor varies dramatically with time, the pressure difference across different floor pairs remains stable. They obtained the relative floor level instead of the actual floor by detecting the number of floor switches using pressure differences. In order to estimate an absolute floor number, Liu et al. [21] deployed a barometer in the building as reference. By calculating the pressure measurement difference between reference barometer and a user’s mobile phone, they obtain the actual floor the user is on, but this method depends on deploying a reference barometer in the environment, which has poor scalability in practice. Ye et al. [22] established a mapping between barometric pressure values and the corresponding floor levels in a crowdsourcing fashion. The mapping is calibrated by encounters between different users. Li et al. [23] obtained the initial barometric pressure by detecting Wi-Fi fingerprints near the entrance of the target building. The limitation of this method is that the Wi-Fi fingerprints near the building entrance must be known. Falcon et al. [24] detected the entrance of the target building by training a neural network using GPS signals. The effectiveness of this method depends heavily on the accuracy and timeliness of indoor and outdoor recognition. Banerjee et al. [25] developed a positioning solution combining Wi-Fi-based localization and barometric altimetry using an unsupervised probabilistic learning method. In summary, due to the fact the barometric pressure is easily affected by weather conditions and ambient air flows, accurate and real-time calibration of a reference pressure is a big challenge for pressure-based methods.

### 2.3. Hybrid Methods

In complex multi-story buildings, adoption of only a single Wi-Fi-based method or barometric pressure-based method cannot produce accurate and robust floor positioning. Several studies have designed hybrid methods to improve floor positioning performance. BarFi [10] exploits the combination of Wi-Fi RSS and barometric pressure for accurate floor positioning. It trains the RSS fingerprint floor map with the aid of a barometer in a crowdsourcing fashion, which is computationally complex. HYFI [9] combines the wireless access point distribution by Bayesian classification and barometric pressure information to determine the floor level. They identify the floor level by discriminating the slow and quick pressure changes. However, the accuracy of using the likelihood-based method in HYFI [9] is low because it only uses the keying-like AP information instead of using the received signal strengths. Li et al. [18] integrated the data from an inertial sensor, magnetometer, and barometer with RSS data through an extended Kalman filter to gain more reliable and smoother floor detection. Reference [26] describes a new barometric altimetry error model, which includes white Gaussian noise and a first order random walk. Then the signals are updated by Wi-Fi RSS measurements using a Kalman filter. Nevertheless, their likelihood to maximum a posteriori (MAP) conversion is loose.

Different from the abovementioned floor positioning methods, our proposed floor positioning algorithm introduces the concept of a confidence parameter into the Wi-Fi-based floor positioning method. The floor positioning result with high confidence obtained by the Wi-Fi-based floor positioning method is used to calibrate the reference barometric pressure in time. To correct occasional floor positioning errors, the HMM is also adopted. Furthermore, we propose an XGBoost- based motion detection method to identify floor switching behavior in real time, which not only can improve floor positioning accuracy, but also can give continuous vertical coordinates once the floor switching motion is detected in the intermediate areas between adjacent floors. 

## 3. Materials and Method

In this section, we outline the overall system and divide it into three parts in detail: BWFP Module, BWFP Augmentation Module and Motion Detection Module.

### 3.1. System Overview

In existing multi-story buildings, such as shopping malls, large office buildings, etc., the geographical environment is often complex, which may contain the intersections of hollow and non-hollow areas, and users may cross multiple floors by various transportation modes (taking elevators, escalators, or going up/down stairs.). This poses a challenge to floor positioning and makes it difficult to rely on a single floor positioning approach. For concision, we define the non-hollow area as a closed area, which means that the adjacent floors are completely separated by concrete slabs.

Benefiting from the various sensors embedded in common smartphones, our system takes both the Wi-Fi [27] fingerprints received by mobile phones and the readings of accelerometers, gyroscopes [28] and barometers [29] as input. Through the combination and improvement of various methods, the floor can be located robustly and accurately.

**Figure 1:**
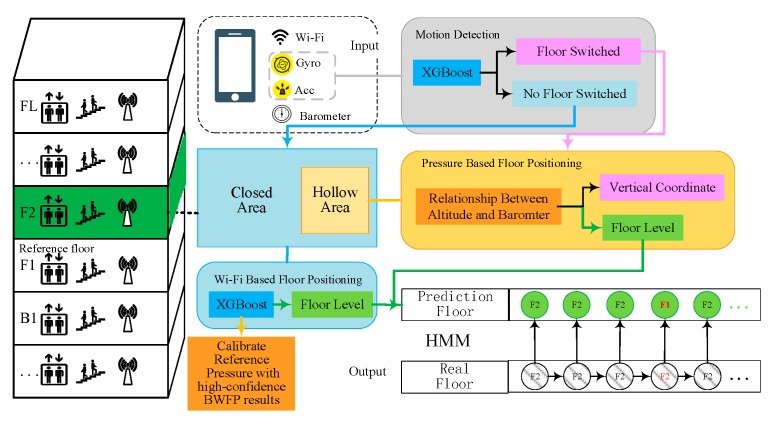
System overview design.

As shown in Figure 1, our Motion Detection Module is designed to accurately detect users’ floor switching motions, including but not limited to going upstairs, going downstairs, taking an elevator up, and taking an elevator down. When no floor transition motion is detected, BWFP is performed to achieve accurate floor positioning in closed areas. However, in hollow areas, it is observed that the Wi-Fi fingerprints between adjacent floors are very similar and it is difficult to accurately localize floors because there is no concrete slab barrier and the wireless signal propagation attenuation is very small. To achieve accurate floor positioning both in hollow and closed areas, we develop Bthe WFP Augmentation Module, which combines BPFP and HMM as a compensation method. BPFP is applied by the mapping between the barometric pressure and altitude. HMM is utilized to correct the occasional floor positioning errors caused by the BWFP or the BPFP method. Once the floor transition event is detected, the vertical coordinates in the intermediate areas between adjacent floors is estimated by the barometric pressure difference between current time and the last time when the motion detection result was changed from the walking motion to the floor switching motion.

In order to accurately localize floors in all complex indoor areas, including hollow, closed areas and intermediate areas between adjacent floors, we must ensure that the right remedy is applied, i.e., the BWFP Module is applied in closed areas, and BPFP and HMM are combined to strengthen floor positioning in hollow areas. However, it is difficult to decide when and which method to apply, because the system does not know whether the user is in a hollow or closed area at any given time. We propose a confidence threshold judgment method to handle this problem. Our system uses the BWFP results with high confidence to opportunistically calibrate barometric pressure. Detailed design will be described in Section 3.3 and Section 3.4 describes how to judge user’s floor switching behavior.

### 3.2. BWFP Module

Due to the barrier of concrete slabs, the access point set and received signal strength of the same AP are remarkably different between adjacent floors in closed areas. By making full use of these characteristics, we can achieve accurate floor positioning in such areas. We model the floor positioning as a supervised multi-classification problem. Each floor is given a different label. The floor with the largest prediction probability is taken as the final floor estimation.

The BWFP method contains two phases: offline training phase and online predicting phase. In the offline training phase, the AP fingerprints collected along all paths on the same floor are given the same label. The number of fingerprints in each floor should be balanced.

Let *L* represents the *p* consecutive floors of a multi-story building and $l_{i}$ represents the $i^{th}$ floor:

$$L=\left\{l_{1},l_{2},\ldots ,l_{p}\right\}$$

and let O be a set of *D* observations as given by:

$$O=\left\{O_{1},O_{2},\ldots ,O_{D}\right\}$$

where $O_{d}\;\left(d\in \left\{1,2,\ldots ,D\right\}\right)$ indicates an observation consisting of m APs and their corresponding RSSI values. 

Considering the fact that the number of scanned APs in different locations on a floor is different, the dimension of observation vector $O_{d}\;$ is dynamic. For convenience, we use $RSS_{r}$ to indicate the received signal strength from the $r^{th}$ AP. 

We introduce XGBoost [12] into the BWFP. The XGBoost classifier combines multiple tree models to build a strong multi-classifier. Before applying the XGBoost classifier, we embed original AP fingerprints in the Euclidean space. In this way, we can define the distances and similarities over features in the Euclidean space. The concrete operation is to run the exponential operation on every $RSS_{j}$ in $O_{d}$, as Equation (1) shows:

(1) $$SV_{O_{d}}=\{\lambda^{RSS_{j}}|j\in \left[1,m\right]\}$$

where λ is the index coefficient. The hyperparameter λ is determined by experiments (in this paper, it is set to 1.035). Since the received signal strengths are negative, the exponential operation maps original signal strengths between 0 and 1, which can speed up the training process. SV is the signal vector after the embedding operation. Next, we attach the ground truth floor as a label to the original data. The set of $SFV_{O_{d}}=\left(SV_{O_{d}};label=l_{O_{d}}\right)$ represent training and testing samples.

Through training, K trees in XGBoost are obtained and the cumulative value of K trees is the estimated floor positioning result as Equation (2) shows:

(2) $${\hat{y}}_{i}=\overset{K}{\underset{k=1}{\sum }}f_{k}\left(SV_{O_{d}}\right),f_{k}\in F$$

where F are all possible trees, $f_{k}\left(SV_{O_{d}}\right)$ is the weight of sample $SV_{O_{d}}$ on the leaves of the $k^{th}$ tree which represents the scores of different floors, ${\hat{y}}_{i}$ is the final estimated floor positioning result obtained by accumulating the $f_{k}\left(SV_{O_{d}}\right)$ of all K trees.

Our loss function is defined as Equation (3) shows and the second element in Equation (3) is the regularization term as Equation (4) shows, where T is the number of leaf nodes, ω is the output score of each leaf node, γ and η are the weight ratios representing preference for two terms. In Equation (3), $y_{i\;}$ represents the ground truth floor, ${\hat{y}}_{i}$. represents the output of classifier and f represents the MSE (Mean Squared Error) between $y_{i\;}$ and ${\hat{y}}_{i}$. *n* is the number of training samples and *K* is the number of trees in the model:

(3) $$Loss=\overset{n}{\underset{i=1}{\sum }}f\left({\hat{y}}_{i},y_{i}\right)+\overset{K}{\underset{k=1}{\sum }}\Omega \left(f_{k}\right)$$

(4) $$\Omega \left(f_{k}\right)=\gamma T+\frac{1}{2}\eta \overset{T}{\underset{j=1}{\sum }}\omega_{j}^{2}$$

Our goal is to balance between MSE and regularization term so as to obtain the optimal model based on a large number of fingerprint training data. Using regularization term can avoid over-fitting by controlling the complexity of model. During the online floor positioning phase, the exponential operation is first applied on the real-timely collected RSSI fingerprints. Then preprocessed RSSI fingerprint is inputted into the pre-trained model to identify the most likely floor on which a user stays.

We introduce the confidence parameter to represent the accuracy of the BWFP method, which is the floor prediction probability using BWFP. Only high confidence floor positioning results are used to calibrate the barometric pressure (which will be detailed in the next section). For example, if the BWFP Module is employed in a four-story building, the four-floor estimation probability is (0.23, 0.28, 0.27, 0.22). We can see that although the prediction probability of F2 is the highest, it is better to discard the floor estimation result and resort to other methods instead. Therefore, we define the maximum probability of floor estimation using BWFP as the confidence. The floor positioning probability can be directly obtained by setting the ‘objective’ to ‘multi:softprob’ in XGBoost. Only the floor estimation with high confidence is accepted. Those floor prediction results with low confidence is discarded. We will detail it in the Section 3.3.

To evaluate the performance of BWFP, we conducted several experiments in four different buildings and their structure is described in Table 1. Building 1 contains large hollow areas from the first floor to the third floor. Building 2 and Building 3 have small hollow interior between adjacent floors. Building 4 is almost entirely hollow from the first floor to the fourth floor. Three Wi-Fi-enabled smartphones (Xiaomi 5s/Huawei Mate20/Huawei Mate9) are used to collect floor fingerprints. We used an application that queried the Android Application Programming Interface (API) for the Wi-Fi fingerprints (0.5-Hz sampling rate for all smartphones). We uniformly collected the AP fingerprints along all paths of each floor in the four buildings. 

We totally collected 5930 training samples from the underground floor to the third floor in Building 1 (1223 samples on the underground floor, 2379 on the first floor, 1551 on the second floor and 777 on the third floor). Among them, 1661 samples were collected using Xiaomi 5s, 1580 samples were collected using Huawei Mate20 and 2689 were collected using Huawei Mate9. There are totally 4058 APs observed in Building 1. Similarly, we collected 3017 training samples from the first floor to third floor in Building 2 (1069 samples on the first floor, 938 samples on the second floor, 1010 samples on the third floor). Among them, 653 samples were collected using Xiaomi 5s, 402 samples were collected using Huawei Mate20 and 1962 samples were collected using Huawei Mate9. There are totally 1102 APs observed in Building 2. We totally collected 812 training samples from the seventh floor to the eighth floor in Building 3 (325 samples on the seventh floor and 487 samples on the eighth floor). There are totally 581 APs observed in Building 3. We totally collected 6995 training samples from the first floor to the fourth floor in Building 4 (2416 samples on the first floor, 1994 samples on the second floor, 1390 samples on the third floor and 1195 samples on the four floor). There are totally 1826 APs observed in Building 3. The samples of Building 3 and Building 4 were all collected by Huawei Mate9. The samples were collected while tester walking all paths within the building. To summarize, there are totally 16754 training samples and it took about 13 min to collect sample per floor on average.

We also collected 6571 test fingerprints of Building 1 about two months after the training samples were collected (1272 samples on the underground floor, 2751 samples on the first floor, 1595 samples on the second floor and 953 samples on the third floor). It is worth noticing that there are 2252 test samples near the hollow areas (825 on the first floor, 798 on the second floor and 629 on the third floor). And the test samples of Building 2, Building 3 and Building 4 were collected five days after the training samples were collected. We totally collected 10849 test samples. The accuracy of floor positioning is defined as the number of correctly identified floor over the total number of test samples. 

Table 2 compares the accuracy of our proposed classification-based BWFP using XGBoost and Bayesian used in HYFI [9] in these four experimental buildings. From this Table, we can find that using the XGBoost based method can obtain better floor positioning accuracy than the likelihood-based method.

In order to verify whether our proposed BWFP model works well in the hollow areas, we established a comparative experiment between hollow and closed areas. We used the pre-trained BWFP model to localize floors in the hollow and closed areas of Building 2, respectively. As can be seen from Table 3, the accuracy in the closed areas reaches nearly 100%, while the average accuracy in the hollow areas is about 95.3%. Besides, the accuracy in F3 is only 89.3%, which is far from meeting the requirements of practical applications.

This shows that even though we use the powerful classifier XGBoost, the positioning accuracy of BWFP in the hollow areas is still unsatisfactory because of indistinguishable Wi-Fi fingerprints between adjacent floors.

### 3.3. BWFP Augmentation Module

The previous section shows that even though we use the powerful XGBoost classifier, the positioning accuracy of BWFP in the hollow areas is still unsatisfactory. In the regions with hollow areas, the Wi-Fi signals are similar and prone to confuse between adjacent floors, so it is difficult to accurately identify floor only by using BWFP. In addition, classification-based BWFP cannot accurately localize floor in the intermediate areas between adjacent floors, which is critical for complete floor positioning. Therefore, we combine BPFP and HMM together as complement. 

The BPFP utilizes the physical relationship [8] between altitude and barometric pressure, as Equation (5) shows, to obtain altitude change for floor positioning:

(5) $$H_{p\;}=\frac{T_{0}}{Lapse}\left[{\left(\frac{P_{s}}{P_{0}}\right)}^{-\frac{Lapse*R}{g}}-1\right]+H_{0}$$

where $T_{0}$ and $P_{0}$ are the temperature and pressure at sea level, respectively.$\;T_{0}$ is 288.15 (K) and $P_{0}$ is 1013.25 (hPa) in the standard atmosphere. Lapse is lapse rate of temperature, R is the universal gas constant and g is the gravitational constant. $H_{0}$ is set to 0 at sea level, and $P_{s}$ represents real-time barometer measurements. However, Equation (5) is only applicable to the calculation under the standard atmosphere condition. For non-standard atmosphere conditions, the obtained altitude using Equation (5) need to be furthermore calibrated as described in [7]. 

For convenience, we use $H_{c}$ to represent the calibrated altitude:

(6) $$H_{c}=H_{p\;}+\xi_{h}$$

where parameter $\xi_{h}$ represents the bias of altitude caused by different air-data conditions which is opportunistically calibrated with high confidence BWFP result. After calculating the calibrated barometric altitude and assuming that the sea level is an approximation of geoid level, we can obtain the altitude with the barometer measurements and vice versa. The BPFP can not only compensate for the unreliable positioning of the BWFP in the hollow areas, but also estimate vertical coordinates in the intermediate area between adjacent floors during floor transitions.

However, barometric pressure is susceptible to environmental factors, such as temperature and humidity change. In modern large-scale buildings, we find that the barometric pressure may be different between areas with a front view towards the sun and a rear view away from the sun. Air conditioning also may affect barometer measurements. In brief, barometer measurements cannot be directly used to identify floor levels without calibration. 

Therefore, the mapping between floor levels and the corresponding barometric pressures must be updated in real time to ensure that the pressure maintained by the system is of confidence and accurate for BPFP. Assuming that each height between adjacent floors is previously known, we only need to maintain a “reference pressure” automatically for a multi-story building during the whole positioning period. We define “reference pressure” as the real-time calibrated pressure of the “reference floor”, which can be any floor within the building (e.g., the first floor in this paper). In the area where the Wi-Fi signals can be distinguished with high confidence, the “reference pressure” is calibrated by the high-reliable result from BWFP. While in the hollow areas, the floor is predicted by using BPFP with “reference pressure”.

**Figure 2:**
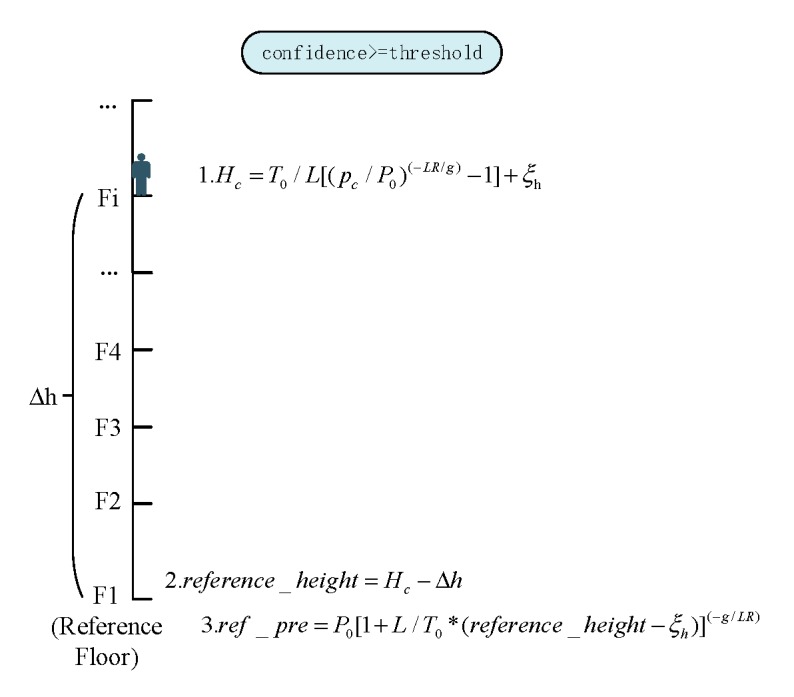
The reference pressure calibration.

**Figure 3:**
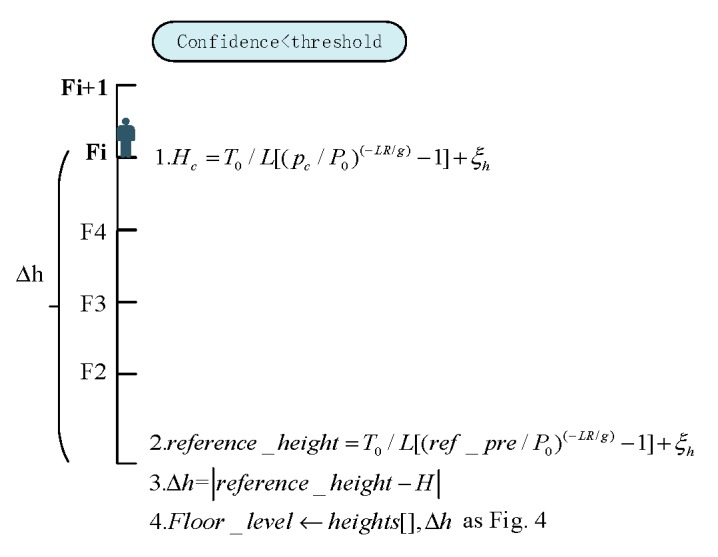
The floor prediction using the BPFP.

As mentioned in Section 3.2, the BWFP Module simultaneously gives the floor positioning confidence corresponding to the floor positioning estimation. We utilize a confidence threshold judgment method to select appropriate floor positioning result of BWFP for BPFP calibration. As a hyperparameter, the floor positioning confidence threshold is selected based on experiments. The higher the threshold, the higher the positioning accuracy requirement for BWFP. By filtering confidence using preset threshold, the right remedy is applied. The relationship between the threshold and the number of BPFP triggers will be discussed in detail in Section 4.1. More specifically, the method includes the following two steps:

1. “Reference pressure” calibration: when the confidence of BWFP exceeds the preset threshold, update the “reference pressure” algorithm as Figure 2 shows. Parameter $p_{c}$ represents the current barometer measurements, and $f_{c}$ represents the floor estimation result obtained by BWFP. We use an array of heights [] to record each height between adjacent floors within a multi-story building. Variable Δh is the height difference between the current floor and the reference floor (the first floor as the reference floor in this paper), which can be estimated based on the parameter $f_{c}$ and heights []. The ref_pre is the calibrated pressure of the reference floor and reference_height is the estimated height of the reference floor.
2. Floor prediction: the “reference pressure” is calculated to predict the floor level. The inference process is shown in Figure 3.

**Figure 4:**
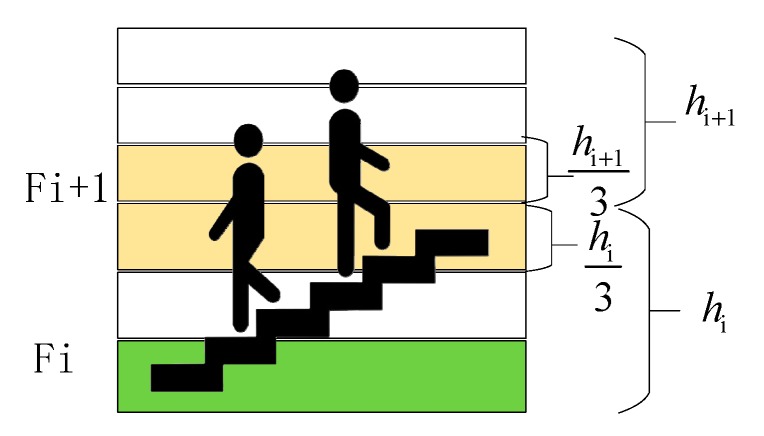
Floor prediction processing.

Considering that the floor level estimated by the abovementioned floor prediction method may not be exactly an integer, and a user may stay in the intermediate areas between two adjacent floors, we design a threshold-based floor detection scheme, as Figure 4 shows.

If $-\frac{h_{i}}{3}\leq h_{gap}\leq \frac{h_{i+1}}{3}$, the user is judged to stay on the $F_{i+1}$ floor. Otherwise, once the floor switching motion is detected, which will be described in the next section, the user is judged to be situated in the intermediate area between adjacent floors and the vertical coordinates are then estimated using Equation (7).

We define:

(7) $$h_{gap}=h_{BPFP}-h_{Fi+1}$$

where $h_{BPFP}$. is the height calculated by the BPFP method, and $h_{Fi+1}$ is the real height of the $F_{i+1}$ floor level. 

Considering that the instability of Wi-Fi signals and the sudden transition between the hollow and closed areas, the occasional floor estimation error may occur. In order to reduce the jump problem, we intuitively leverage the Hidden Markov Model (HMM) [30] to correct the final floor prediction. Based on the Markov hypothesis, HMM has the temporal characteristics of recording historical information. We define our HMM model as Equation (8) shows:

(8) λ=(A,B,π)

where *A* is the transition probability, which is the probability of moving from one floor to another. It is calculated from the statistical analysis of user behavior training datasets. *B* is the emission probability and is obtained from the prediction confusion matrix. Parameter *π* is the initial state probability vector, which is obtained by probabilistic statistics from the user behavior training dataset.

We regard the floor estimation obtained by BWFP or BPFP as the observation and the ground-truth floor as the hidden state in the Markov chain. We utilize the uncertain floor estimation obtained by BWFP or BPFP to speculate the floor which the user most likely stays on. It is equivalent to the decoding problem in HMM. Given the model and floor observation sequence, the HMM reveals the hidden (real) floor level using Viterbi method [31]. We abbreviate real floor as rf, prediction floor as pf. The floor estimation is modeled as maximum optimization problem as Equation (9) shows.

(9) $$\left(rf_{1},rf_{2},\ldots ,rf_{N}\right)=argmax\;P(rf_{1},rf_{2},\ldots ,rf_{N}|pf_{1},pf_{2},\ldots ,pf_{N})$$

According to the Markov hypothesis, Equation (9) is equivalent to:

$$\left(rf_{1},rf_{2},\ldots ,rf_{N}\right)=argmax\;\overset{N}{\underset{i=1}{\prod }}P(rf_{i}|pf_{i})P(pf_{i}|pf_{i-1})$$

To make a conclusion, we propose a BWFP augmentation with BPFP and HMM. The augmentation mainly includes two parts: firstly, the BPFP is used to strengthen the hollow areas and the intermediate areas between adjacent floors where the BWFP cannot accurately provide floor positioning result. In this case, only “reference pressure” needs to be maintained. On the other hand, HMM is used to correct the occasional errors of floor positioning. The overall performance is evaluated in Section 4.

### 3.4. Motion Detection Module

Most existing floor positioning methods can only estimate floor level, which cannot provide services while users are moving in the intermediate area between adjacent floors. As a critical judgment condition, as Figure 1 shows, we first detect users’ motion using inertial sensor measurements. Once the floor transition motion is detected, the vertical coordinate of user is continuously estimated. Otherwise, the floor is estimated by the BWFP Module or the BPFP Augmentation Module.

When a user moves between different floors (going upstairs/downstairs, taking elevator up/down), the changing patterns of barometric pressure, acceleration and angular velocity are different from those when user walks along the same floor. This provides inspiration for us to accurately detect floor switching motion. 

However, due to the pressure drift with environment factors, it is very unreliable to calculate the height change directly using barometer measurements as described in [32]. Therefore, we propose to detect users’ motion using accelerometer and gyroscope readings. We regard motion detection as a binary classification problem, which includes floor switching motion and non-floor switching motion. Floor switching motions include but not limited to going upstairs/downstairs, taking elevator up/down. Once the floor switching motion is detected, then we calculate the barometric pressure difference between current time and the last time when the motion detection result was changed from the non-floor switching motion to the floor switching motion. Combined with Equation (6) in the previous section, the user’s vertical coordinates can be calculated.

**Figure 5:**
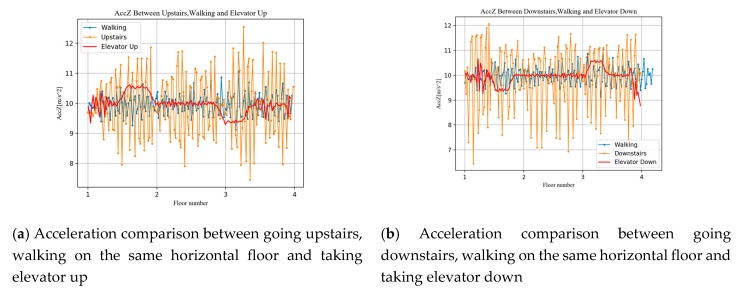

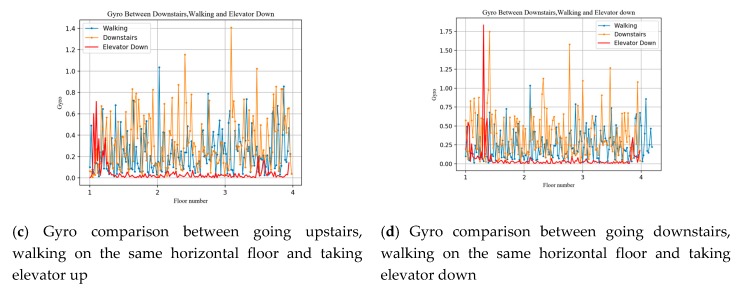
Acceleration comparison and gyro comparison between different vertical motion.

Theoretically, acceleration can characterize a sudden change of user motion, such as an overweight or underweight state during floor switching behavior. The gyroscope can detect the turning motion between the floors when the user goes upstairs or downstairs. In order to verify the feasibility of this idea, we collected the measurements of accelerometer and gyroscope during walking, going upstairs, going downstairs, taking elevator up, and taking elevator down. The experimental results are shown in the Figure 5. Note that non-floor switching motion measurements are collected on the same floor and we stretch the data for comparison. As can be seen from Figure 5a,b different motions produce different acceleration change modes, such as a sharp drop and increase while taking the elevator up or down. And the acceleration also demonstrates a large degree of oscillation while going upstairs or downstairs compared to walking on the same horizontal floor. As shown in Figure 5c,d the gyro data are slightly different under different motion modes, and we expect the classifier to judge the user’s motion pattern by distinguishing the change pattern of acceleration and gyro.

Considering that the device attitude fluctuation with different holding modes and user walk produces various noise, we perform the coordinate transformation from the carrier coordinate system (b-system) to the navigation coordinate system (n-system) for all sensor data to obtain uniform sensor features, which is free of different holding modes and user walk. The coordinate system transformation from the carrier coordinate system to the navigation coordinate system [33] is shown as follows:

(10) $$C_{b\left(m\right)}^{n}=C_{b\left(m-1\right)}^{n}C_{b\left(m\right)}^{b\left(m-1\right)}$$

(11) $$C_{b\left(m\right)}^{b\left(m-1\right)}=\left[I+\frac{\sin\Delta \theta_{m}}{\Delta \theta_{m}}\left(\Delta \theta_{m}\times \right)+\frac{1-\cos\Delta \theta_{m}}{{\left(\Delta \theta_{m}\right)}^{2}}{\left(\Delta \theta_{m}\times \right)}^{2}\right]$$

(12) $$\Delta \theta_{m}=\int_{t_{m-1}}^{t_{m}}\omega_{ib}^{b}d\tau \approx \omega_{ib}^{b}T_{s}\;\Delta \theta_{m}=\left|\Delta \theta_{m}\right|$$

where $\omega_{ib}^{b}$ is the angular velocity obtained by the gyro measurements. $\Delta \theta_{m}$ is the angle at which the carrier rotates from time m-1 to m. $C_{b\left(m\right)}^{b\left(m-1\right)}$ is the transformation matrix of b-system from time m-1 to m. $C_{b\left(m\right)}^{n}$ is the attitude matrix and should be updated in real time based on the output of gyro.

Motion detection is divided into offline training phase and online motion detection phase. In the offline training phase, to improve floor transition detection accuracy and reduce the detection delay, a sliding window with 50% data overlap is used to collect 2.56 s of accelerometer and gyro measurements (called a data frame). Then all of training data frames are labelled according to the corresponding user motion. Data frames encapsulated based on different sensor data are carefully observed from four different levels of granularity, i.e., statistical features, time domain features, peak and segment features and frequency domain features. They are combined into the final frame feature vector.

More specifically, we extract a total of 46 features based on frame data as Table 4 shows. “vertical” represents Z-axis measurements and “horizontal” represents the modulus of X-axis and Y-axis measurements. In this module, we also employ the XGBoost method to fit the collected training data and extract the features from the real-time readings for motion detection.

As a vital decision factor, the accuracy of motion detection should be ensured. Similar to Section 3.2, the HMM is also used to correct the occasional motion detection errors. We collected training data with 2774 floor switching data (including going upstairs and downstairs, taking elevator up and down) and 3761 walking data on the horizontal floors using three different mobile phones (Huawei Honor V1, Huawei Mate9, Xiaomi MIX2).

The sensor sampling frequency is set to 100 Hz, i.e., for every 10 ms, an acceleration sample and a gyroscope sample of mobile phones are collected. Next, a data sample (256 pieces of data) was constructed every 2.56 s to construct a feature vector.

From the simulation experiment, the importance of each feature for XGBoost classifier was obtained. The red mark features in Table 4 are the most important features. It can be seen that the FFT amplitude of acceleration and angular velocity can differentiate stride span and frequency when users walk on the same floor or a vertical movement happens.

**Figure 6:**
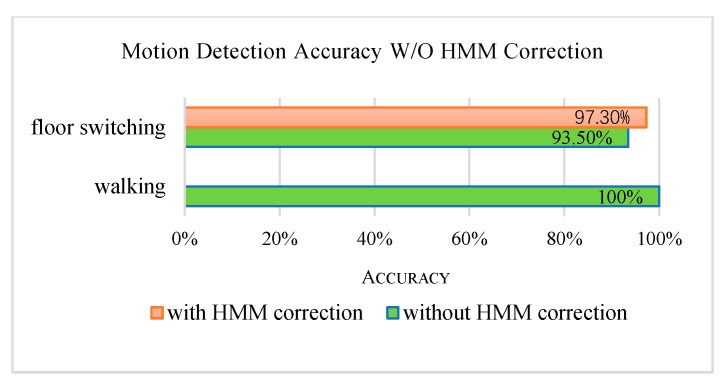
Motion detection accuracy W/O the HMM correction.

On the real-time testing stage, two testers walk on the horizontal floors and take floor switching activities for 5 min in Building 2, respectively. The motion detection accuracy is shown in Figure 6.

## 4. Experimental Results and Analysis

### 4.1. Augmentation BWFP with BPFP Using HMM

The above experiments confirm that purely using the BWFP cannot meet the application requirements both in the hollow areas and in the closed areas. And purely pressure-based method cannot identify floor level directly without calibration. Therefore, we combine BWFP with BPFP, and further use HMM to filter out occasional floor estimation errors. In this part, the effectiveness of our proposed method and each component is tested.

**Figure 7:**
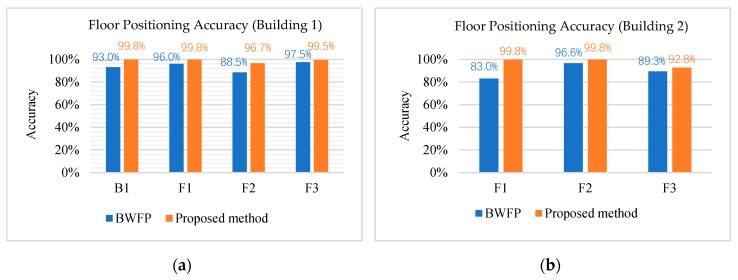
Floor positioning accuracy in (**a**) Building 1 and (**b**) Building 2.

As shown in the Figure 7, the total test results in Building 1 and Building 2 confirm that the proposed method can improve the accuracy of floor positioning compared with only using a single floor positioning method.

**Figure 8:**
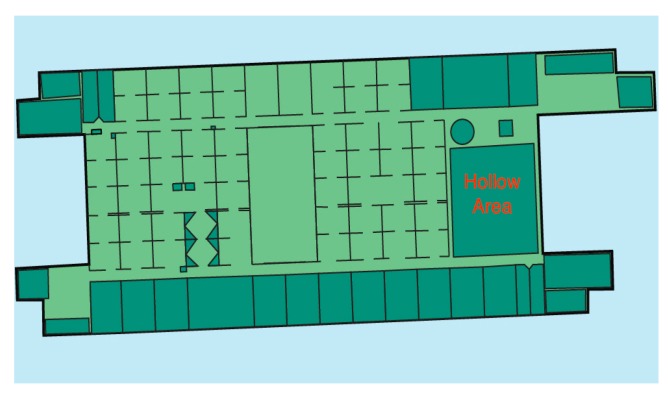
The floor plan of F7 in Building 3.

In more detail, we tested the relationship between the threshold value in the calibration process and the number of times BPFP is triggered, as well as the accuracy. Trigger tests were carried out on the seventh floor (F7) of Building 3. The floor plan of the F7 is shown in Figure 8. The hollow area is located at the right of the whole map, which is marked in red. It is worth noting that the HMM component was turned off during the trigger tests.

**Figure 9:**
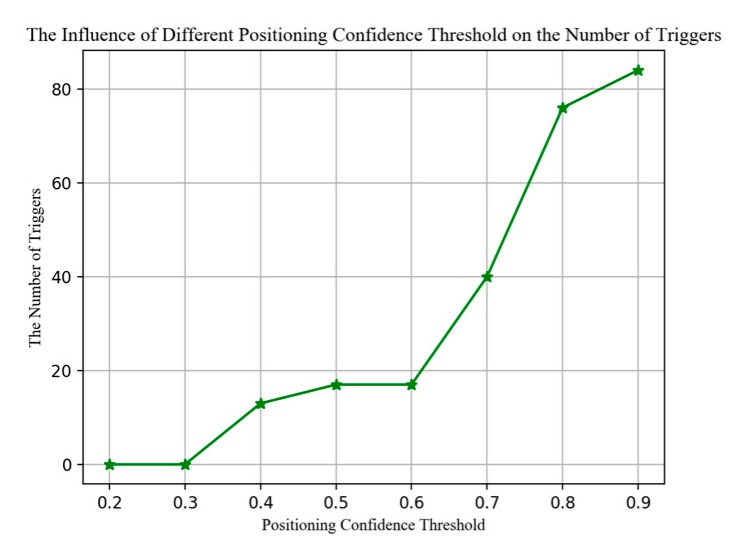
The influence of different floor positioning confidence threshold on the number of triggers.

**Figure 10:**
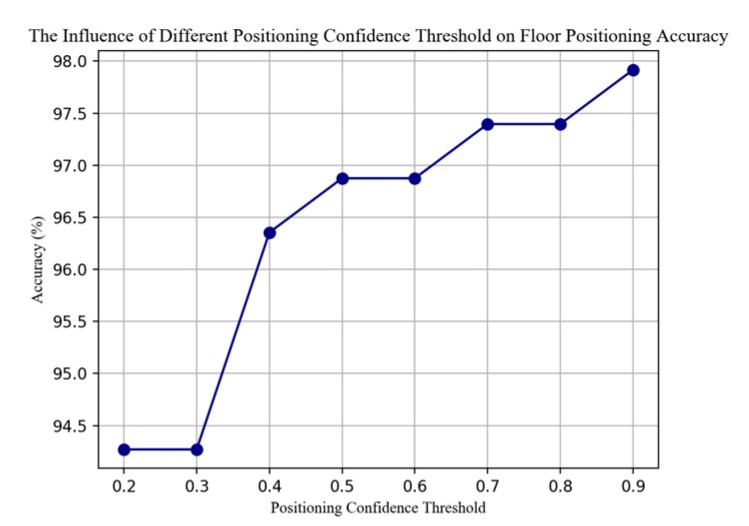
The influence of different floor positioning confidence threshold on the floor positioning accuracy.

As shown in Figure 9, the higher the threshold, the more times BPFP is triggered. In our tests, the accuracy of floor positioning also increases with the increase of the threshold Figure 10 benefiting from the high accuracy of BPFP in short time and in confined space. However, in practical applications, excessive dependence on BPFP is unreliable because of the long-term instability of barometric pressure. For trade-off, we choose a threshold of 0.7 in this paper.

**Figure 11:**
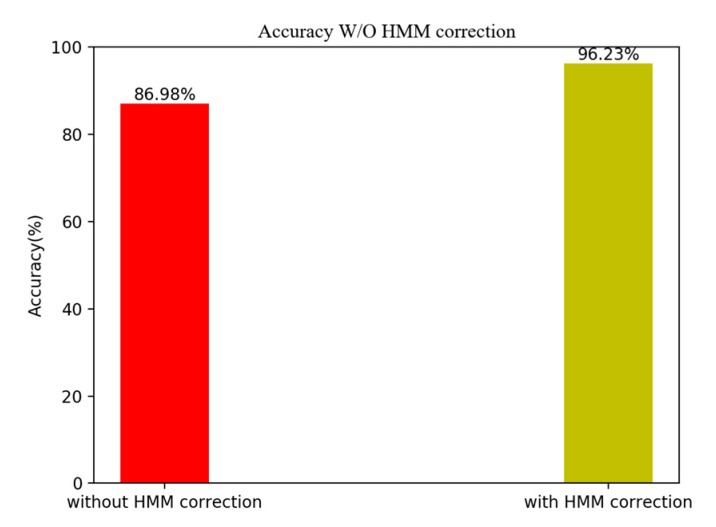
Accuracy without and with HMM correction.

To correct occasional error of floor positioning caused by wireless noises and barometric noises, we introduce the HMM to filter the floor positioning results obtained by the BWFP and the BPFP. As Figure 11 shows, due to the use of historical information, the accuracy has been improved after performing the HMM correction.

Moreover, we compare our proposed method with several other floor identification methods mentioned in [17]. From Figure 7a, the average accuracy of our proposed method is 99.2%. Table 5 shows our proposed hybrid floor positioning method outperforms other single sensor methods and fusion methods with the same sample datasets. The parameter k in k-NN [13] test is assigned to 8, 9, 10, 11 respectively and the optimal accuracy is obtained when k is 11. In the ANNs [15] test, we define two layers of 50 neurons per layer and choose sigmoid as the activation function. In FLD [17] test, we calculating corresponding weight vector by combining the two pairs in the sample sets. The initial value of parameter $S_{w}$ and $S_{b}$ in [26] is assigned to 0.2 m and 0.1 m.

### 4.2. Motion Detection and Positioning in the Intermediate Areas Between Floor

**Figure 12:**
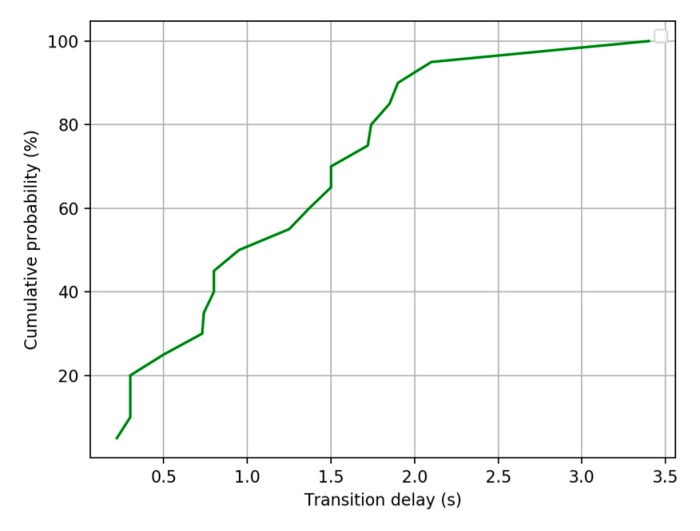
The cumulative probability of transition delay.

We conducted several experiments in Building 2 with three floors. During testing, three testers (U1, U2, U3) walking on four paths (F1 → F2, F2 → F3, F3 → F2, F2 → F1). Through multiple groups of tests, it can be seen that cumulative probability of switching delay within 2 s exceeds 90% as Figure 12 shows. The switching delay is defined as the time difference between the time when user arrives at the new floor and the system is updated to the new floor.

**Figure 13:**
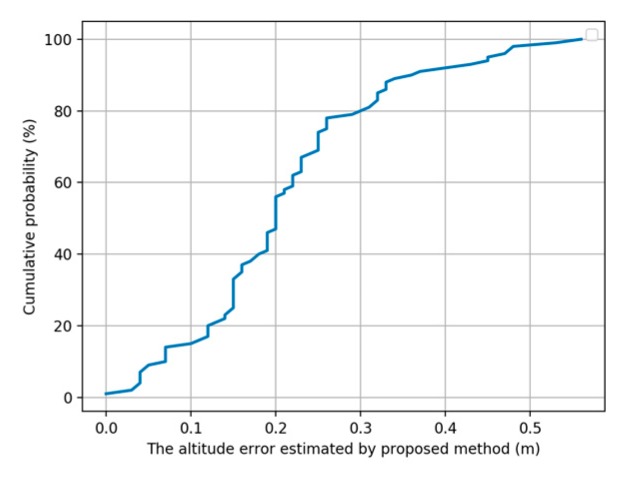
The cumulative probability of the altitude error estimated by our proposed method in the intermediate areas between floors.

We also tested the performance of our proposed method in the intermediate areas between floors. The tester moves from F7 to F8 in Building 3 with holding the phone and stays at one quarter, one-half, three quarters of the intermediate areas for 10 s. We collected 50 sets of data and the cumulative probability of the altitude error estimated by our proposed method in the intermediate areas between floors is shown as Figure 13. The average height estimation error using our proposed method in the intermediate areas is about 0.26 m and the probability of altitude error within 0.5 m exceeds 90%, which confirms that our proposed floor positioning method can provide continuous and accurate vertical coordinates within multi-story buildings.

### 4.3. Caculation Complexity

We compared the training time and testing time using different classification model to evaluate the calculation complexity of our proposed algorithm. Table 6 lists the time cost of different classification models.

From Table 6 we can see that, although Bayesian model requires less training time than the XGBoost model, its accuracy in actual testing is far less than that of the XGBoost model. On the other hand, longer offline training time cost does not affect the user application online. Therefore, as a trade-off measure, it is more appropriate to choose XGBoost as the BWFP classifier. In actual test, XGBoost takes less time than Bayesian method. By dividing each feature into blocks and sorting the blocks, the XGBoost adopts the parallel computing method to search for the best splitting point, which greatly reduces the testing time [34].

## 5. Discussion and Conclusions

In this paper, we have proposed a multi-mode fusion floor positioning architecture, which embraces the BWFP method using XGBoost, BWFP augmentation with BPFP using HMM, and motion detection. Through the integration of multiple methods, the accuracy and delay which affect the floor positioning field have been greatly improved. Furthermore, our proposed method can provide continuous vertical coordinates when users walk up/down stairs or takes an elevator in the intermediate area between floors. By adopting a confidence threshold, we built a bridge between BWFP and BPFP, and used HMM method to filter out the occasional floor positioning errors. The proposed motion detection method makes our floor positioning to a higher level and achieves a precise and robust milestone. We evaluated our proposed algorithm in four complex indoor environments. The evaluation results demonstrate that the floor positioning accuracy can reach 99.2% and the probability of switching delay within 2 s exceeds 90%, which outperforms other existing floor positioning methods and can meet the real-time requirements of floor positioning.

As future work, we have identified some minor problems in our proposed method, especially the extra overhead caused by the selection of many hyperparameters, such as the threshold of switch between BWFP and BPFP, the pre-trained emission probability of HMM, etc. These values require labor-intensive data collection for each new building. We will try crowdsourcing methods to reduce the need for expert work.

## Figures and Tables

**Table 1:** The structure of four buildings to evaluate the BWFP algorithm.

Building_ID	Building_Name	Building_ Location	No_of_Floors	No_of_Underground_Floors	No_of_Aboveground_Floors
1	Bantian J	Shenzhen, China	4	1	3
2	Bantian H	Shenzhen, China	3	0	3
3	ICT	Beijing, China	12	0	12
4	Teaching Building of BUPT	Beijing, China	4	0	4

**Table 2:** The floor estimation accuracy of BWFP with Bayesian model in four different buildings (%).

Model	Building 1	Building 2	Building 3	Building 4
Bayesian	87.6	99.3	97.1	82.6
XGBoost	95.2	99.7	99.9	96.4

**Table 3:** The accuracy comparison of BWFP between hollow and closed areas in Building 2 (%).

Areas	F1	F2	F3	Mean
Hollow Areas	99.8	96.0	89.3	95.3
Closed Areas	99.8	99.3	99.8	99.7

**Table 4:** Motion detection features selected for the XGBoost.

Features (a Total of 46)		
**Accelerometer**	***Vertical***	Mean, std, var, median, min, max, range, iqr
	***Horizontal***	Mean, std, var, median, min, max, range, iqr
	***Modulus***	Mean, std, var, median, min, max, range, iqr, kurtosis, skewness, rms, integral, double integral, correlation, FFT
**Gyro**	***Modulus***	Mean, std, var, median, min, max, range, iqr, kurtosis, skewness, rms, integral, double integral, correlation, FFT

Note: more detailed description of the features seen in the Appendix A.

**Table 5:** Floor positioning accuracy comparison with other methods.

Method	Accuracy (%)
Proposed method	99.2
FLD [17]	93.7
k-NN [13]	81.3
ANNs [15]	90.6
Fusion [26]	93.8

**Table 6:** Comparison of different algorithms training and testing time.

Model	Training Time (ms)	Testing Time (μs)
Bayesian	1022	28075
XGBoost	2368	438.1

## Appendix A

**Table A1:** Motion Detection Feature.

Feature	Equation	Illustration
Mean	$\frac{1}{n}(\sum_{i=1}^{n}x_{i})=\frac{x_{1}+x_{2}+\ldots +x_{n}}{n}$	Represents trends in a set of data sets
Std	$\sigma =\sqrt{\frac{\sum_{i=1}^{N}{\left(x_{i}-\bar{x}\right)}^{2}}{N}}$	A measure that is used to quantify the amount of variation or dispersion of a set of data values.
Var	$\sigma^{2}=\frac{\sum_{i=1}^{N}{\left(x_{i}-\bar{x}\right)}^{2}}{N}$	Measures how far a set of (random) numbers are spread out from their average value.
Range	$R=\max\left(x_{i}\right)-min\left(x_{i}\right)$	The difference between maximum and minimum
Iqr	Interquartile range	The IQR is a measure of variability, based on dividing a data set into quartiles.
Kurtosis	$Kurt\left[X\right]=E\left[{\left(\frac{X-\mu }{\sigma }\right)}^{4}\right]$	A measure of the "tailedness" of the probability distribution of a real-valued random variable.
Skewness	$\gamma =E\left[{\left(\frac{X-\mu }{\sigma }\right)}^{3}\right]$	A measure of the asymmetry of the probability distribution of a real-valued random variable about its mean.
RMS	$x_{rms}=\sqrt{\frac{1}{n}(x_{1}{}^{2}+x_{2}{}^{2}+\ldots +x_{n}{}^{2}}$	In the evaluation of experimental results, the errors must be positive or negative relative to the average value. RMS can better reflect the discreteness of experimental results errors by eliminating the symbolic effect when the error is squared.
Integral	$E\left(f\right)=\int_{a}^{b}\omega \left(x\right)f\left(x\right)dx$	In our experiments, the acceleration integral represents the velocity and the angular velocity integral represents the rotation angle.
Double Integral	$A\left(x\right)=\int_{z}^{b}[\int_{\varphi_{1\left(x\right)}}^{\varphi_{2\left(x\right)}}f\left(x,y\right)dy]dx$	Displacement is expressed by double integral of acceleration
